# Doing research in non-specialist mental health services for children and young people: lessons learnt from a process evaluation of the ICALM (Interpersonal Counselling for Adolescent Low Mood) feasibility randomised controlled trial

**DOI:** 10.1186/s40814-023-01427-7

**Published:** 2024-01-23

**Authors:** Thando Katangwe-Chigamba, Jamie Murdoch, Paul Wilkinson, Viktoria Cestaro, Carys Seeley, Eirini Charami-Roupa, Tim Clarke, Aoife Dunne, Brioney Gee, Sharon Jarrett, Andrew Laphan, Susie McIvor, Richard Meiser-Stedman, Thomas Rhodes, Lee Shepstone, David A. Turner, Jon Wilson

**Affiliations:** 1 Norwich Clinical Trials Unit, Norwich Medical School, University of East Anglia, Norwich, UK; 2 School of Life Course and Population Sciences, Kings College London, London, UK; 3 School of Clinical Medicine, University of Cambridge, Cambridge, UK; 4 Paediatric Psychology, Addenbrookes Hospital, Cambridge, UK; 5 Research and Development, Norfolk and Suffolk NHS Foundation Trust, Norwich, UK; 6 Norwich Medical School, University of East Anglia, Norwich, UK; 7 Public Health, Suffolk County Council, Ipswich, UK; 8 Children and Young People’s Services, Suffolk County Council, Ipswich, UK; 9 Department of Clinical Psychology and Psychological Therapies, Norwich Medical School, University of East Anglia, Norwich, UK

**Keywords:** Depression, Low mood, Early help, Process evaluation, Qualitative

## Abstract

**Background:**

The rising prevalence of adolescent mild depression in the UK and the paucity of evidence-based interventions in non-specialist sectors where most cases present, creates an urgent need for early psychological interventions. Randomised controlled trials (RCTs) are considered the gold standard for obtaining unbiased estimates of intervention effectiveness. However, the complexity of mental health settings poses great challenges for effectiveness evaluations. This paper reports learning from an embedded process evaluation of the ICALM RCT which tested the feasibility of delivering Interpersonal Counselling for Adolescents (IPC-A) plus Treatment as Usual (TAU) versus TAU only for adolescent (age 12–18) mild depression by non-qualified mental health professionals in non-specialist sectors.

**Methods:**

A qualitative mixed methods process evaluation, drawing on Bronfenbrenner’s socioecological model to investigate key influences on trial delivery across macro-(e.g. policy), meso-(e.g. service characteristics) and micro-(e.g. on-site trial processes) contextual levels. Data collection methods included 9 site questionnaires, 4 observations of team meetings, policy documents, and 18 interviews with stakeholders including therapists, heads of service and managers. Thematic analysis focused on understanding how contextual features shaped trial implementation.

**Results:**

The ICALM trial concluded in 2022 having only randomised 14 out of the target 60 young people. At a macro-level, trial delivery was impacted by the COVID-19 pandemic, with services reporting a sharp increase in cases of (social) anxiety over low mood, and backlogs at central referral points which prolonged waiting times for mild cases (e.g. low mood). An interaction between high demand and lack of capacity at a meso-service level led to low prioritisation of trial activities at a micro-level. Unfamiliarity with research processes (e.g. randomisation) and variation in TAU support also accentuated the complexities of conducting an RCT in this setting.

**Conclusions:**

Conducting a RCT of IPC-A in non-specialist services is not feasible in the current context. Failure to conduct effectiveness trials in this setting has clinical implications, potentially resulting in escalation of mild mental health problems. Research done in this setting should adopt pragmatic and innovative recruitment and engagement approaches (e.g. creating new referral pathways) and consider alternative trial designs, e.g. cluster, stepped-wedge or non-controlled studies using complex systems approaches to embrace contextual complexity.

**Trial registration:**

ISRCTN registry, ISRCTN82180413. Registered on 31 December 2019.

**Supplementary Information:**

The online version contains supplementary material available at 10.1186/s40814-023-01427-7.

## Key messages regarding feasibility

*Uncertainties existed regarding the feasibility*

• The paucity of evidence-based interventions in non-specialist and voluntary sectors where most adolescents with mild depression present and receive support from non-mental health professionals creates an urgent need for suitable early psychological interventions. Uncertainties existed regarding the feasibility and acceptability of conducting randomised controlled trials (RCTs) in this setting, including recruitment (of participants and therapists) and randomisation.

*Key feasibility findings*

• Presenting findings from the embedded process evaluation of the ICALM (Interpersonal Counselling for Adolescents (IPC-A) Low Mood) feasibility trial, which only randomised 14 out of a target of 60 eligible young people to receive IPC-A plus Treatment As Usual (TAU) or TAU, we provide insight into contextual challenges of conducting RCTs in this setting. Key feasibility barriers include (1) high demand and lack of capacity, leading to a lack of research prioritisation; (2) low familiarity with research procedures, especially randomisaton; (3) variation of service models and unclear service specifications; and (3) a lack of oversight of service coordination.

*Implications of the feasibility findings for the design of the main study*

• Conducting a RCT of IPC-A in non-specialist services is not feasible in the current context.

Research in non-specialist mental health services needs to adopt more pragmatic and innovative recruitment (e.g. creating new referral pathways) and engagement approaches, that can be continually reviewed to account for rapidly evolving service contexts. Researchers should also consider alternative trial designs, such as cluster randomisation, stepped-wedge designs or non-controlled studies using complex systems approaches to embrace the complexity of contextual conditions.

## Background

Depression is a common mental disorder experienced by young people (YP) worldwide, with prevalence rates for major depressive disorder (MDD) in adolescents ranging from 11 to 20% [1, 2]. Depression in adolescents is a major risk factor for suicide [3] and predicts a range of adverse outcomes in adulthood [4–6]. Despite high MDD prevalence in this age group, research highlights even higher mild/sub-threshold depression rates [7]. In the United Kingdom (UK), the prevalence of YP experiencing low mood or depression increased from 15% in 2010 to 41% in 2021[8]. Mild depression is associated with significant personal and public health consequences [9] and is a strong predictor for future onset of MDD [10]. Considering that half of mental health problems are established by the age of 14 [11], intervention targeted at younger people has been estimated to result in greater personal, social and economic benefits [12].

In the UK, most mental health services for YP are commissioned through a Tiered system: Tier 1 represents early intervention and prevention services; Tier 2 involves Early Help and targeted services; Tier 3 consists of specialist Child and Adolescent Mental Health Services (CAMHS); and Tier 4 constitutes a specialised units for assessment and treatment of severe mental health problems. The vast majority of adolescents seeking treatment for depression have a mild disorder [13] and are unlikely to meet treatment thresholds for specialist Tier 3 services. Whilst there has been a recent increase in targeted tier 2 services, referral pathways are often unclear, meaning YP receive help from a large variety of services. As such YP often receive support from non-qualified mental health professionals in local authority/third sector/voluntary agencies who usually have no formal training in delivering evidence-based treatments. In addition, there is a dearth of evidence-based interventions in non-specialist contexts where most cases of mild depression present [14–16]. Randomised controlled trials (RCTs) remain the gold standard method for obtaining unbiased estimates of interventions effectiveness [17, 18]. However, to our knowledge, no RCTs for mild depression have taken place in Tier 1 and 2 services.

Given the paucity of evidence-based interventions in non-specialist contexts where most cases of mild depression present, Interpersonal Counselling for Adolescents (IPC-A) was developed as a potential treatment for mild depression in these services. This is a 3–6 session structured therapy which focuses on the links between interpersonal relationships and depressive symptoms. A single-arm pilot study conducted in Early Help services suggested significant reductions in depressive symptoms and high acceptability for YP and practitioners [19]. In addition, discussions with stakeholders indicated the intervention to be a good fit for the services. Therefore, the ICALM (Interpersonal Counselling for Adolescent Low Mood) study sought to feasibility test the delivery of a full-scale RCT of IPC-A delivered in non-specialist community services by youth mental health workers without core professional training, compared to Treatment as Usual (TAU). The study aimed to contribute to the evidence base in line with the Department of Health’s Framework for Mental Health Research, which recommends that ‘research should focus on early intervention and involve organisations beyond traditional mental health services, including local authorities and the voluntary sector’ [20].

The full protocol of the ICALM study, which planned to randomise 60 eligible YP, has been published elsewhere [21]. Trial commenced in January 2020 but encountered unanticipated contextual challenges, including the COVID-19 pandemic. As such recruitment started later in September 2020 and ended later than predicted in February 2022, having only randomised 14 participants. The trial thus provided insufficient data to answer key feasibility questions.

Drawing on the MRC guidance on developing and evaluating complex interventions [22], the feasibility RCT was accompanied by a mixed-methods process evaluation to generate an understanding of intervention implementation. Process evaluations nested within trials and exploring implementation of the intervention and trial processes can provide valuable insight into why interventions fail or have unexpected consequences, and identify contextual factors associated with variation in outcomes [23]. Due to the difficulties experienced with implementing the ICALM trial in this setting, the trial team and funder agreed that the process evaluation should focus on understanding the challenges of conducting RCTs in the Tier 1 and 2 mental health services. In this article, presenting findings from the embedded process evaluation of the ICALM trial, we provide detailed insight into barriers faced during trial delivery, with an emphasis on reporting key learnings on what made conducting research in this setting particularly challenging. The findings have important implications for researchers planning to conduct future evaluations in non-specialist mental health and community services.

## Methods

### The ICALM trial

The aim of the ICALM trial was to explore the feasibility of a full-scale RCT of the effectiveness and cost-effectiveness of IPC-A by (1) assessing the feasibility and acceptability of trial procedures including recruitment and randomisation; (2) exploring the delivery of IPC-A and Treatment as Usual (TAU), (3) evaluating the extent of contamination in the control arm; and (4) investigating indications of interval benefits of IPC-A over TAU in mild depression [21]. The full quantitative results of the trial will be published elsewhere. A qualitative process evaluation was incorporated in the trial to explore feasibility in depth; this paper focuses on the findings of this aspect of the study.

### The intervention

IPC-A is a brief manualised psychological intervention (3–6 sessions) [24], derived from Interpersonal Therapy for Adolescents (IPT-A). It is a 3–6 session structured therapy focusing on links between interpersonal relationships and depressive symptoms. Whilst IPT-A is a NICE-recommended first-line treatment for adolescents with moderate to severe depression designed for delivery by qualified mental health professional within specialist CAMHS (Tier 3), IPC is designed for clients with mild depression; and our previous pilot study demonstrated that it can be delivered by non-specialist mental health professionals [19].

### Target population

Eligible participants were YP (aged 12–18 years) accessing participating services via standard referral pathways seeking help for low mood (as the primary presenting difficulty) and able to provide written informed consent or, for under 16 years, written informed assent and parent/guardian consent. Participants were randomised in a 1:1 ratio to receive either IPC-A plus TAU or TAU only.

To deliver IPC-A, youth mental health workers needed to have no core professional mental health qualification (e.g. psychiatrist, psychologist) [25] and needed to receive a two-day training, followed by weekly supervision [19].

### The setting

The trial was conducted in two English counties: Norfolk and Suffolk, where Tier 1 and 2 services (including services for mild depression) are largely provided by non-specialist services, as the severity of illness is generally below treatment thresholds for National Health Service (NHS) CAHMS (Tier 3). Whilst not all Tier 1 and 2 services are specifically commissioned to provide mental health support, YP with mental health issues often present in all services. Non-specialist support is provided by voluntary sector organisations, publicly commissioned independent counselling organisations and County Councils.

### The process evaluation

The initial objectives of the embedded process evaluation of the ICALM trial were to (a) provide a description of how IPC-A and TAU were delivered; (b) assess implementation and theoretical fidelity to the IPC-A model; (c) observe how delivery is shaped by the context of differing service models; (d) identify any harms arising from treatment; and (e) establish the extent and source of any contamination. However, recruitment challenges triggered a request from the funder to refocus process evaluation objectives with greater emphasis on understanding barriers and enablers to running RCTs in this setting. This article specifically addresses this new objective.

### Procedures

The qualitative process evaluation adopted an ethnographic approach using multiple qualitative methods including interviews, observations, policy reviews and a site profile questionnaire, to address the set objectives. Data collection occurred concurrently with the feasibility trial.

Interviews were conducted with a broad range of stakeholders including non-mental health professionals, service managers, head of services, clinical leads and senior practitioners. Due to high staff turnover within participating services, participants were conveniently sampled. Informed written consent to participate in interviews was obtained separately to consent for the main study. Interview topic guides explored systematic challenges with delivering mental health services for YP, policy implementation, and views on the role of early interventions such as IPC-A and conducting research in this setting. Interviews with non-mental health professionals and service managers also focused on understanding trial implementation challenges, staff resource allocation, TAU, and perceptions of the IPC-A intervention.

Observations of staff training and meetings involving the research team and service managers were conducted throughout the trial and aimed to understand key concerns and challenges faced by participating sites with regards to trial and intervention delivery. Profile questionnaires were collected from participating sites to understand the broader service context, including usual support for YP with mental health needs and current challenges impacting on the delivery of usual support.

### Data analysis and synthesis

Drawing on concepts from Bronfenbrenner’s socioecological model [26] to provide structure for reporting contextual barriers and enablers, the analysis was structured across macro (the system), meso (the organisation) and micro (the team) levels.

All interview/focus group data and observational data were transcribed verbatim and thematically analysed [27] then mapped across macro-, meso- and micro-levels. To further understand how participating sites were organised to provide services at a meso-level, we analysed questionnaire responses to describe service characteristics and identify key differences between the services and initial barriers/enablers to trial implementation. To further develop key influences at a macro-contextual level, policy documents were reviewed in conjunction with discussions with stakeholders and members of the research team [27]. The analysis was iterative, moving between data collection and analysis to test emerging concepts. For example, when we observed managers expressing challenges with study processes or protocols during meetings, we followed this up during interviews to gain a more in-depth understanding of these challenges and the implications for trial delivery. The final synthesis of the data considered how national policy and other relevant contextual features at a macro-level shaped the organisation and delivery of non-specialist mental health services at a meso-level, and how this interacted with the ICALM intervention at a micro-level.

## Results

First, we provide a summary of the ICALM trial findings, followed by the process evaluation results consisting of (1) a description of the participating organisations and (2) a report on macro- and meso-contextual features of non-specialist mental health services and how they were made salient during trial implementation at a micro-contextual level.

### The ICALM feasibility trial: summary of results

The full report of the quantitative findings of the ICALM trial will be reported elsewhere. The Consort diagram for the feasibility trial has been provided in Additional file 1. In total 16 out of the target 60 participants were randomised, two of whom withdrew. Despite considerable efforts from the study team, including joining weekly site meetings and holding discussions with commissioners (in Suffolk) to understand pathways, identify barriers and find new recruitment routes into the study, recruitment was significantly lower than anticipated. However, participant retention was high with 85.7% of participants reaching 23-week follow-up. Fidelity of the intervention was satisfactory, with all sessions rated as adherent. Due to the study being underpowered, there was no indication of a statistically significant difference between IPC-A and usual support.

### Process evaluation results

Thirteen teams across Norfolk and Suffolk took part in the feasibility trial: six in Suffolk and seven in Norfolk. Data collected included 11 interviews with stakeholders, seven interviews with therapists (six IPC-A and one TAU), nine questionnaires and four observation field notes of meetings. Characteristics of interview participants are described in Table 1. To retain anonymity, organisations and participants have been given an ID.

**Table 1:** Characteristics of interviewed participants

**Stakeholder ID**	**Organisation ID**	**Role**
01	Site-10_Central Referral Point	Clinical Nurse Specialist
02	Site-04_Wellbeing Service	Team Manager
03	Site-08_NHS Community Trust	Clinical Lead
04	Site-01_Early Help Team	Team Manager
05	Site-02_Early Help Team	Team Manager
06	Site-09_Family Support Team	Head of Service
07	Site-04_Wellbeing Service	Team Manager
08	Site-07_ Wellbeing Service	Wellbeing Clinical Manager
09	Site-05_Charity	Senior Practitioner
10	Site-03_Early Help Team	Acting Manager
11	Site-06_Charity	Senior Emotional Wellbeing Practitioner
**Therapist ID**	**Organisation ID**	**Study arm**
TAU-01	Site-06_Charity	TAU only Therapist
IPC-01	Site-08_NHS Community Trust	IPC-A trained Therapist
IPC-02	Site-09_Family Support Team	IPC-A trained Therapist
IPC-03	Site-02_Early Help Team	IPC-A trained Therapist
IPC-04	Site-09_Family Support Team	IPC-A trained Therapist
IPC-05	Site-01_Early Help Team	IPC-A trained Therapist
IPC-06	Site-05_Charity	IPC-A trained Therapist

### Description of services

An overview of the characteristics of the nine sites with completed profile questionnaires is provided in Additional file 2. Participating services consisted of Early Help teams, charities, family support teams, a wellbeing service, a community NHS Trust, and a School Nursing team. Early Help teams and Family support teams funded by social care through county councils to address family or social issues including low mood in YP. Charities can be commissioned by health or social care or run entirely by donations and in this context were supporting YP with a variety of social and mental health challenges through youth work or counselling. Wellbeing teams, Community Trusts and School Nursing teams are all NHS funded to provide either tier 2 targeted interventions or/and universal services.

Service providers estimated 40–90% of their referrals to involve some mental health difficulty, in particular low mood and anxiety. Only four services offered targeted short-term (6–8 sessions) interventions (e.g. CBT-informed interventions) for YP experiencing mild-moderate mental health problems such as anxiety and low mood. Targeted services consisted of staff with and without professional mental health qualifications, including children’s wellbeing practitioners (CWPs), primary mental health workers (PMHWs), cognitive behavioural therapists (CBT) and counsellors. Within these services, only CWPs, who do not have a core professional mental health qualification, delivered IPC-A.

Non-targeted services were set up to address emotional difficulties (albeit within a social framework). However, despite mental health difficulties being commonplace for YP presenting to their services, process evaluation findings indicated that non-targeted services (Site-1-3_Early Help teams, Site-09_Family Support Teams and the Site-08_community NHS Trust) did not offer any formal mental health support (apart from IPC-A as part of ICALM) but primarily depended on onwards referral to targeted services where mental health difficulties were identified or recognised to form a primary part of the presentation. This is a crucial finding since sites were recruited with the proviso that they were already providing some form of mental health support to YP with low mood.

Recent stats have shown that approximately three quarters of our referrals are around young people’s mental health [SPQ-03, Site-03_Early Help]

There’s no other intervention [apart from IPC-A] that’s specifically focussed on mental health…[SPQ, Site-02_Early Help Team]

Non-targeted services primarily consisted of staff with no professional mental health backgrounds including family support practitioners (FSP), Young Person’s Workers (YPW) and resilience and emotional health practitioners. FSPs and resilience and emotional health practitioners came from various backgrounds including education and social care. YPWs were primarily Joint Negotiating Committee qualified or had a Level 3 qualification in Health and Social Care.

### Macro-level contextual features of mental health services impacting trial delivery

At a macro-level, delivery of ICALM was largely impacted by the COVID-19 pandemic, structural challenges at referral points and policy implementation challenges.

#### The COVID 19 pandemic

The study commenced (Jan 2020) approximately three months before the first COVID-19 lockdown in the UK. The impact of the pandemic and ensuing restrictions on mental health services and the ICALM study are summarised in Additional file 3a. Services reported a sharp increase in cases and a shift in presentation of cases from low mood to (social) anxiety, germ-phobic behaviour, and agoraphobia, creating major challenges in identifying suitable cases for IPC-A. In addition, services experienced high levels of staff sicknesses and absences, significantly reducing capacity. This created unprecedented challenges, with participating sites having to manage an increased demand at reduced capacity, whilst navigating new ways of delivering interventions virtually. These challenges eventually led to the withdrawal of one team and a complete halt in Norfolk teams referring to the study, making it necessary to recruit further teams.

#### Structural challenges at central referral points

During the ICALM trial, the Central referral point in Suffolk, which receives *approximately 9000 referrals a year and ranks amongst the top three in the country for referrals* [Stakeholder-01, Site-10_ Central referral point], was experiencing huge backlogs. These backlogs prolonged waiting times for green cases which were eligible for IPC-A, due to prioritisation of red and amber cases (green = low risk, e.g. low mood or anxiety; amber = moderate risk, e.g. suicidal ideation with lesser intent/planning; red = significant risk, e.g. actively suicidal).

The target for a green, which will be a low mood…would be 28 days. We are not there at the moment. We are at about five months. But when I started six months ago, we were at 11 months [Stakeholder-01, Site-10_Central referral point]

Apart from the COVID-19 pandemic, stakeholders attributed the backlog to pre-existing structural challenges (see Additional file 3b) at front line referral points (i.e., primary healthcare and school personnel). This included a lack of knowledge of (and support for) front-line staff regarding mental health conditions and support pathways, leading to inconsistencies and inadequate information on referral forms, subsequently causing significant delays in referral processes.

We’ve also found… they’re asking for an ADHD assessment, and it says, ‘ADHD diagnosed’ and has a date. So, we’re like, well why are they diagnosed? No, what it’ll be is a SENCo [Special Education Needs Coordinator] has said to a parent, ‘I think your kid’s got ADHD’ They go and tell the GP. The GP thinks it’s a diagnosis and records it on their record. That cannot be removed, and they may not even have it [Stakeholder-01, Central referral point]

The ICALM trial was therefore embedded in a context whereby referral services were focused more on urgent cases and overcoming structural challenges, and less on green cases which were potentially eligible for IPC-A.

#### Mental health policy

Relevant mental health policy reviewed for documentary analysis is set out in Additional file 3c. Capacity and resource allocation challenges, stemming from a disconnect between workforce strategy and policy objectives about meeting different YP needs, were a major barrier to implementing the ICALM trial. A key challenge of implementing the mental health policies has been a lack of specificity about how to allocate funds to different (staff) resources. The lack of clarity about how to operationalise policy at a service level is most recently evident in the NHS Long Term Plan [28], which has objectives to improve access by providing a 0–25 service and 24/7 crisis service. However, there is a lack of specificity about how to provide and implement the services, numbers of staff required and how senior clinical roles will be deployed to supervise new staff/trainees.

Problems of capacity are not isolated within mental health services and other service sectors are also overwhelmed with demand for care. Consequently, YP have experienced being referred across multiple mental health, voluntary, social care and health services, driven by individual providers placing boundaries around which types of problems they treat and don’t treat in order to manage demand. This was done unilaterally, without consultation across the system. Within this context, the challenge of asking mental health services to implement the IPC-A intervention and feasibility trial becomes clear.

A lot of the time it’s [volume of referrals] driven by process and system issues, and the fact that services are overstretched and they’re trying to move people on a lot of the time, and not rightly or wrongly [Stakeholder-01, Central referral point]

A central policy agenda has been to reduce waiting times into specialist mental health services [29]. However, this agenda set within a context of overwhelming demand has arguably created a public perception that mental health services provide the gold standard set of interventions to YP that are worth waiting for. This suggests that greater effort is required to promote the philosophy of mental health as ‘everyone’s business’ [30]. ICALM’s objective of training non-mental health professionals from across the system in IPC-A was to make interventions accessible at whatever points in the system YP present with emotional problems whether in education, social care or within the wider community, hereby upskilling the wider workforce and enabling mental health to become everyone's business and within their skill set to offer interventions. This could have the effect of taking the pressure off mental health service and potentially normalising mental health interventions within the wider community. It is possible that provision of IPC-A at this level could be cost-effective if it were demonstrated to be (1) more effective than existing interventions; and/or (2) lead to fewer referrals to more specialist (and expensive) CAMHS services.

### Meso-level contextual features of participating services

The meso-contextual features of participating services and their impact on ICALM are described below and summarised in Additional file 3d. At a service level the findings highlight an interaction between enormous demand, a lack of capacity, unclear service specifications and a lack of oversight of service coordination.

#### Overwhelming demand leading to increase in complexity of cases

In most services, long waiting times led to the deterioration of green cases, consequently rendering interventions targeting ‘mild’ presentations, e.g. IPC-A, less helpful. The trial team, learning of the large number of potentially suitable cases being stuck on a ‘green’ list, endeavoured to set up a fast-track service from the Central referral point, so that potentially eligible ‘green’ rated cases could be referred straight into ICALM. The fast-track service also aimed to reduce the referral of ‘green’ cases which had since increased in severity and were thus not suitable for IPC-A. However, despite services showing great flexibility, due to late implementation, process barriers and difficulties engaging with YP who had been on the green list for so long, the initiative was unsuccessful, recruiting no additional YP.

#### Restrictive and/or unclear service specifications and treatment pathways

Structured evidence-informed mental health interventions were not seen as core elements of Early Help services, making it difficult to introduce IPC-A. This was despite the Early Help threshold matrix stating that *Children and Young People displaying signs of deteriorating mental health and episodes (e.g., low mood/mild depression) should be classed as level 2 and are suitable for Early Help or targeted support services *[31]. This recommendation differed from what was happening at a service provision level whereby Early Help teams referred all cases with mental health difficulties, including low mood, to targeted mental health services or needed to work alongside them.

Mental health is a very small part of what we do but is also very common. We would work alongside any families experiencing any mental health problem, we wouldn’t necessarily take the lead on the mental health part [SPQ, Site-01_Early Help Team]

This disharmony between the service remit and practice, in part driven by reduced capacity and increased demand, was also reported by targeted services. However, targeted services appeared to be more flexible and confident in supporting YP that would not ordinarily fall within their remit.

Our service spec is only considered out of date because of the pressures on the system…it’s structured as an early intervention service spec…but we’ve just morphed into a service that adapts and accepts that we need to extend our boundaries a little bit … there’s nowhere else for these patients…to go into the system [Stakeholder-02, Site-04_Wellbeing Service]

The lack of clarity in service remits and referral/assessment processes, which some stakeholders described as a ‘system level dysfunction’, presented a degree of challenge for the ICALM trial in establishing eligibility of cases.

#### Capacity challenges

Capacity challenges in the mental health sector have been a long-standing issue [32], exacerbated by the COVID-19 pandemic. Factors driving the insufficient capacity were identified as lack of funding and recruitment/retention challenges. A major challenge for targeted services was the poor retention of CWPs, who are often employed on short terms contracts. Thus, high turnover was a significant barrier in the progression of ICALM where most staff that had received training to deliver IPC-A either left the services or moved onto other roles. Capacity challenges especially affected targeted services, as only a small proportion of their staff members do not have a prior mental health qualification (CWPs), hence eligible to train and deliver IPC-A.

Only 5(ish) out of 80 didn’t have prior mental health qualification. 4 or 5 of the 6 trained [in IPC-A] then left the organisation; leaving one available to do the intervention. And it was hard because we’ve got so many referrals coming in, their main job…was to do assessments...so…we didn’t have the capacity… [Stakeholder-11, Site-06_Charity]

### Micro-level study implementation barriers

#### Research prioritisation

At a micro-level, process evaluation findings highlighted a lack of research prioritisation compounded by enormous demand for the services and a lack of capacity. Capacity challenges, exacerbated by the COVID-19 pandemic, led to managers having to prioritise the wellbeing of staff and continuity of service by not participating in activities that were seen as ‘extra’, including research (i.e., ICALM). This was despite services having earlier identified IPC-A as helping them be more effective.

We just had to be quite strict about how we prioritised our time management during the pandemic. And anything additional we just had to be really clear that we weren’t going to take on, for our own wellbeing and therefore the continuity of the service. Nobody has had the capacity I think, or few people have had the capacity to do extra anything, and a trial ends up feeling like extra [Stakeholder-09, Site-05]

#### Randomisation

The lack of familiarity with research processes, in particular randomisation, was a barrier to initial study set-up. Training and meeting observations noted that practitioners struggled with the concept of randomisation due to concerns of putting YP forward for the ICALM study, knowing that they might not be allocated to the IPC-A arm. It should also be noted that some sites (e.g. Site-03_Early) had been involved in the single-arm IPC-A pilot study, which stakeholders felt had been easier and more straightforward compared to the RCT.

Practitioners are struggling with randomisation and the idea that young people might get IPC-A or they might not. [Prompt: Do you think that your team’s involvement with the single arm study might have influenced this?] Yes, that was much simpler because they all got the intervention [SPQ-03, Site-03_Early Help]

The capacity challenges faced by some targeted services posed practical challenges in establishing two study arms within the same team. As such, some targeted services collaborated to either deliver IPC-A or TAU. For example, Site-04_Wellbeing Service referred their YP randomised to TAU to Site-07_Charity.

#### Establishing treatment as usual

One of the objectives for the process evaluation was to describe TAU for low mood within participating services. Our findings, highlighting a wide variation in the support offered to YP, with some Early help teams having no standard intervention for low mood, accentuate the complexities in setting up RCTs in this setting.

As a team, we shouldn’t really be dealing with anything more complex than low level low mood or anxiety, anything more complicated should go to a clinician [mental health professional] but at the moment, we’re not even in trained in how to support the low mood and anxiety stuff [SPQ-02, Site-02_Early Help]

At the beginning of the study, the research team’s understanding was that low mood falls within the remit of both Early help and targeted services. However, when under pressure from excessive demand, Early Help sites resorted to screening out low mood, as this was no longer seen as their core business.

If the young person’s issue is only low mood and they are randomised to the TAU then we refer them back to wellbeing specialists e.g., school nurses and wellbeing services and /GP practices. If they are randomised to IPC-A then whatever case they have should be within the Early Help remit i.e., low mood… and if the other issues can be dealt with by the Family Support Practitioners, then that’s ok [Meeting with Site-02_Early Help]

#### Variation of service models and implementing ICALM

ICALM adopted a model that aimed to recruit and train Early Help practitioners, who are not professionally trained but work broadly with mental health issues. The rationale behind exploring a variety of services was to take a pragmatic approach in support of provision for mild depression, thus obtaining transferability of findings. IPC-A, being relational in its focus, should theoretically fit with the philosophy of the Early Help approach. However, whilst all services supported YP who could potentially be eligible for IPC-A, each service model presented unique challenges to the conduct of the trial. For example, despite Early help teams reporting a significant proportion of YP with low mood, the lack of training and expertise in identifying suitable cases presented challenges for recruiting.

We don’t have any training in supporting young people with their mental health. We do not take on cases which include mental health difficulties without support from wellbeing services…. [SPQ-02, Site-02_Early Help]

Although I think the intervention [IPC-A] could work well in sort of settings where there are mental health presentations, but the workers are not mental health practitioners…there needs to be support with the triage to ensure that the right young people are selected for that intervention [Stakeholder-09, Site-05_Charity]

Family Support Teams also presented a unique challenge in implementing an individualised intervention such as IPC-A in a service dedicated to supporting families as a unit. Practitioners at this site felt that offering IPC-A was not feasible when FSPs are dealing with the whole family to develop an intervention plan that has more to do with safety than therapeutic need. Practitioners felt that IPC-A could work better if offered at the ‘tail end of a sequence of interventions’ when YP are in a more stable situation and environment.

There was also potential for missing some eligible cases as not all teams in the same organisation were involved or had the capacity to deliver IPC-A. Despite repeated attempts by the research team to raise awareness, some managers carrying out assessments remained unaware of the referral criteria for the ICALM study, and hence were not looking out for eligible participants.

You don’t necessarily need them [managers] all to be involved but they all need to understand that when a case comes in, they’re looking at it for potential allocation under those parameters to specific workers… you have three managers for each locality…so it may have been lots of cases came into family support that would have been suitable, but they went to different managers, and ICALM managers never had sight of them in the first place. [Stakeholder-06, Site-09_Family Support Team]

## Discussion

The ICALM study is the first to feasibility test a RCT of IPC-A delivered by staff without core professional training compared to TAU in UK non-specialist mental health services for YP. The results of the study revealed that conducting a RCT in this setting is not feasible in the current context. This paper, reporting results of a process evaluation embedded in the ICALM trial, highlights key contextual barriers to conducting RCTs in this setting.

At a system level, the findings highlight complex and uncoordinated referral systems [33], and unclear workforce strategies for operationalising policy objectives. This in turn creates organisational level challenges (e.g. low staffing levels, high staff turnover, and high caseloads), which, when compounded by the COVID-19 pandemic, significantly impacted capacity of staff to undertake research. The system- and organisational-level barriers, although not isolated to the ICALM trial [34, 35], have a number of implications for researchers and commissioners seeking to identify and develop evidence-based interventions in research-naïve settings such as non-specialist services.

Firstly, the lack of a research culture and familiarity with research processes poses engagement barriers and elucidates the lack of evidence-based guidance in this setting. This results in local interpretations of best practice, especially for clinical presentations such as low mood. Despite various interventions being available (e.g. family interventions, behavioural activation, counselling or IPC-A) there appears to be no clinical rationale or strategy for choosing one intervention over another [36]. This approach to intervention provision within this context poses a challenge for local commissioners to establish processes and frameworks for identifying and implementing evidence-based interventions. Secondly, the disharmony between service remits and practice, which tended to be determined by capacity challenges, makes it difficult to introduce new interventions such as IPC-A. This is compounded by staff who consider mental health as not their core business, despite seeing a large proportion of YP with mental health challenges. Finally, intervention evaluations conducted in settings where routine practice is to screen out all mental health cases, are likely to face significant recruitment problems. This is particularly pertinent if the research is reliant on a similar task-shifting approach adopted in the ICALM trial [37], which relied on staff likely to lack experience identifying eligible cases.

There is, therefore, a need to develop effective unifying workforce capacity building and retention strategies, which can enable services to deliver appropriate interventions across the range of severity and problem types that are commonly presented. As part of a wider workforce capacity-building strategy, developing a culture which recognises the value of developing, evaluating, and implementing evidence-based interventions will facilitate future research [35]. More practically, research naïve teams should be introduced to research processes, including randomisation, early but on a smaller scale before exposing teams to large RCTs. In addition, there is a need to educate clinical services about clinical research and for commissioners to integrate research into service specifications. Staff training and supervision provided as part of research projects needs to prioritise the development of confidence and skills to identify eligible participants alongside the intervention delivery. Training in identification could be provided more widely from the start of the trial and include whole teams not just those who are involved in delivering interventions. Whilst both were included in our training and supervisions, in retrospect, the prominence given to the latter contributed to teams not feeling confident in the crucial task of identifying eligible participants.

The failure of RCTs of complex interventions in mental health settings that showed promising results in exploratory trials [19] is not isolated to the ICALM study [21]. With recent reports highlighting the increase in prevalence of mild depression, failure to conduct RCTs to estimate intervention effectiveness and cost-effectiveness of new interventions such as IPC-A has clinical implications for the management of low mood for young people in UK settings where most cases present.

Further, the problems encountered in ICALM may not be limited to non-specialist mental health services. A recent feasibility study testing a behavioural activation intervention for the management of depression for YP delivered by clinicians in specialists’ settings, has so far been unsuccessful in recruiting cases of low mood [34]. The single-arm study, although demonstrating preliminary evidence for effectiveness and successfully recruiting 33 participants, only identified YP with moderate to severe depression. Such findings further highlight the need to address recruitment challenges for cases of low mood caused by complex and uncoordinated referral systems [38], where YP with low mood end up either on a waiting list or falling through gaps where there are no services to meet their needs. Midgely et al. have recently reported a single-group, uncontrolled design pilot of an Internet-Based Psychodynamic Treatment for Adolescents with Low Mood in the UK, seeking to increase accessibility and address system barriers to YP accessing mental health support such as those faced identified by the ICALM trial (e.g. a lack of service provision and long waiting times) [39]. The study successfully recruited 23 adolescents and found a statistically significant reduction in depressive symptoms. However, with the study having been conducted within a world-leading mental health charity and recruitment having taken place primarily through schools and social media, it cannot be easily generalised to UK settings where most YP with low mood present. In addition, a successful pilot single-arm study does not guarantee a successful RCT, as we have found.

The successful conduct of effectiveness trials in this non-specialist setting requires collaborative working between researchers, services and commissioners to create more streamlined services for YP and promote closer working between Early Help/Social Care teams, primary mental health services (Tier 2 services) and Secondary Services (Tier 3 CAMHS). Researchers need to form collaborations with key players in referral processes (e.g. primary care and referral hubs), early in the research development stages, to improve the identification of, and access to, potentially eligible participants [40]. In this trial, although significant efforts were made to form collaborations with some leverage, considerations of bringing influencers from services on as co-collaborators on trials to influence further investment in research might be required. Researchers also need to prepare and plan for service change that could happen between early trial discussion and the trial ending, ensuring regular communication with commissioners so that the successful running of a trial is considered in service reconfiguration.

These challenges to trial design raise questions about whether any controlled study will feasibly yield meaningful results about effectiveness within such highly variable and changing environments. An examination of final reports of effectiveness trials published by the UK National Institute for Health Research showed that between 2008 and 2014, a greater failure rate of trials of complex interventions in mental health (80%, 12 of 15) than in other medical fields (68%, 23 of 34) [41]. Therefore, instead of persisting with RCTs as the perceived gold standard for producing high-quality evidence, evaluating interventions in mental health settings may require study designs which attempt to embrace the complexity of contextual conditions rather than reduce and control them. These may include study designs which draw on complex system perspectives to understand how both the intervention and wider health system need to adapt to improve outcomes over time [42, 43].

Finally, this research makes the following recommendations for consideration by researchers, service providers, commissioners and research funders:

### Recommendations for researchers

I. Research in non-specialist mental health services needs to adopt more pragmatic and innovative recruitment (e.g. creating new referral pathways) and engagement approaches based on ‘what will work best’ for this setting to address research questions. Such approaches should be developed in collaboration with key stakeholders and in consideration of priorities for potential service providers. For example, creating novel ways to incentivise services to participate and support research.
II. Recruitment strategies should be developed early in the research design process but must be continually reviewed to account for rapidly evolving service contexts. This would require ethics committees to have systems which can expedite amendments to recruitment strategies, ensuring they support the need of research to adapt to changing service contexts.
III. In considering the study design, researchers should have a good understanding of the usual support. If usual support is variable and/or non-existent, or if staff capacity is low to enable the delivery of two study arms, researchers should consider study designs which provide greater flexibility in how usual support is incorporated as a point of comparison to intervention (e.g. cluster RCTs, non-controlled studies using complex systems approaches).
IV. In research naïve settings, researchers should have a good understanding of staff competencies, and where appropriate, provide training on identifying suitable cases in addition to intervention delivery.
V. Researchers should consider alternative trial designs, such as cluster randomisation or stepped-wedge designs, to reduce barriers from reluctance around individual-level randomisation. If using individual randomisation, researchers should provide staff training/support regarding the rationale for randomisation. Research is needed to test the comparative acceptability of alternative randomisation methods.

### Recommendations for commissioners, service providers and policy makers

I. There is a need for Tier 1 and 2 service providers to define and update current service specifications, with consideration given to developing universal assessment criteria. Updated specifications should integrate the undertaking of research to facilitate building a research culture.
II. Commissioners should consider mapping out core offers and resources across the Tier 1/2 services in order to: identify gaps in current service provision; ensure services are joined up within the wider system and have the capacity to support YP; and, using results from this mapping exercise, consider how best to fill service gaps.
III. Commissioners and service leads should consider developing a framework for identifying and delivering evidence-based interventions [44]. Building such a research culture and incorporating appropriate frameworks may increase the success of recruitment to and implementation of RCTs in this setting.
IV. Systems should support the competency development of their workforce through offering training/development opportunities relating to clinical research and the implementation of evidence-based interventions.

### Recommendations for research funders

I. Funders should ensure that researchers planning to conduct trials in this setting have considered the multiple barriers identified by this study; and assess whether they have appropriate plans to ameliorate these.
II. Funders should consider alternative funding models. For example, direct research funding of treatment costs (in both treatment arms) may ameliorate the problems arising from low service capacity, and resultant reluctance to prioritise research. Whilst not a standard part of funding for NHS RCTs, it may be essential for RCTs to function outside NHS settings.

## Conclusion

Process evaluation of the ICALM feasibility study provides insight into contextual challenges influencing the lack of evidence-based interventions in non-specialist sectors. Failure to conduct RCTs to estimate the effectiveness of new interventions such as IPC-A in non-specialist services has clinical implications for the management of low mood. Successful implementation of evaluations in non-specialist sectors calls for researchers to work collaboratively with services to develop innovative solutions to complex referral pathways and increase site engagement by having shared aims. Research conducted in this setting would need to adopt more pragmatic study designs (e.g. cluster RCT, stepped wedge design, complex systems research) that take into account the wide variation of research and clinical experience, as well as service remits.

### Supplementary Information

**Additional file 1.** Consort diagram for the ICALM feasibility trial.

**Additional file 2.** Characteristics of participating early help and targeted services.

**Additional file 3.** The impact of the COVID-19 pandemic on the services and the ICALM trial.

## Data Availability

All supporting data has been included as Additional files.
