# Supplementary figures and images for: Doing research in non-specialist mental health services for children and young people: lessons learnt from a process evaluation of the ICALM (Interpersonal Counselling for Adolescent Low Mood) feasibility randomised controlled trial
Source: Pilot Feasibility Stud. 2024 Jan 23;10:14. doi: 10.1186/s40814-023-01427-7 (PMC10804551; doi:10.1186/s40814-023-01427-7)

**Additional file 1: Consort diagram for the ICALM feasibility trial**


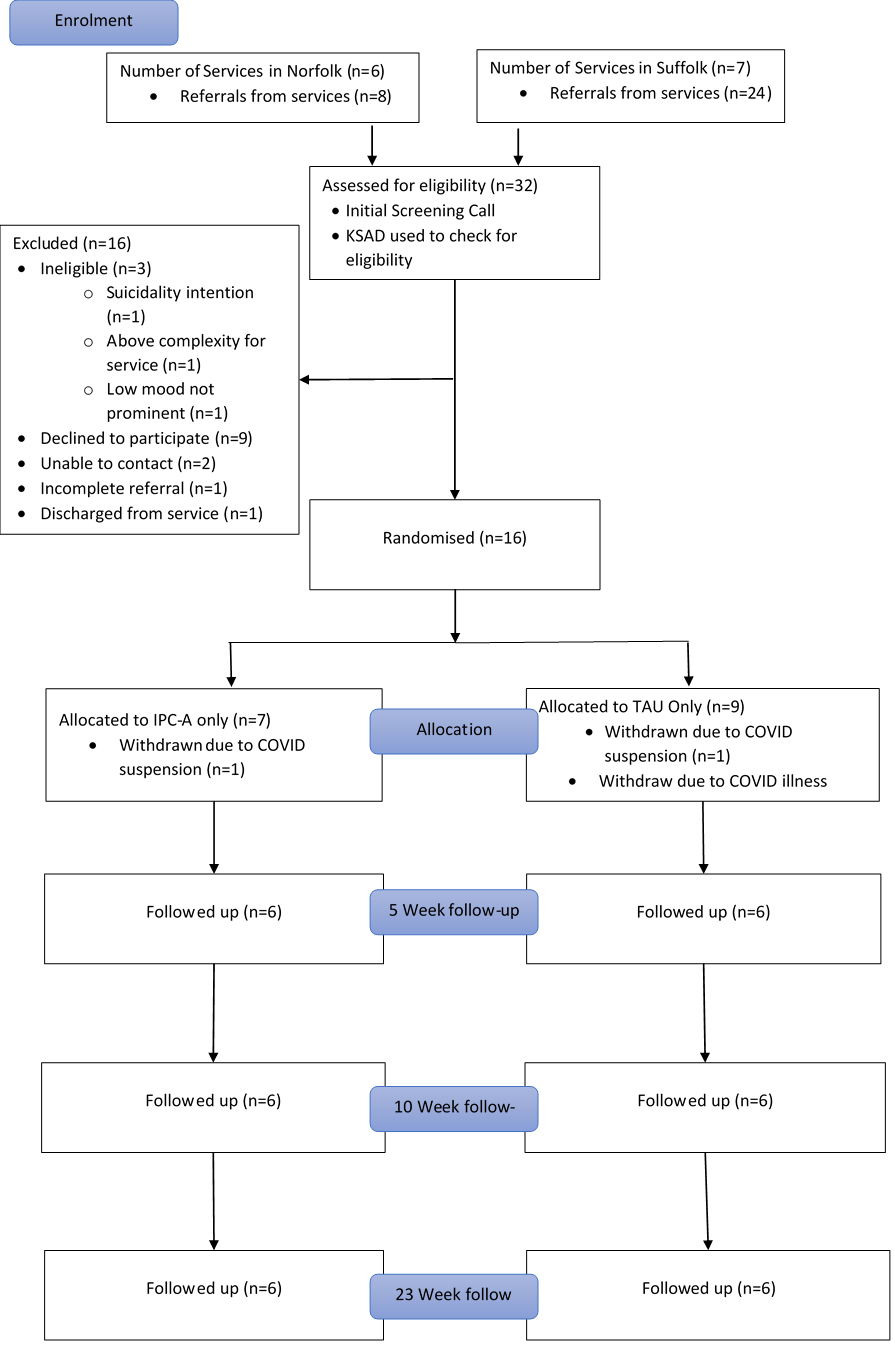

Supplement: Supplementary file 1 — Additional file 1. Consort diagram for the ICALM feasibility trial. [file 40814_2023_1427_MOESM1_ESM.docx]
