# Supplementary material for: Doing research in non-specialist mental health services for children and young people: lessons learnt from a process evaluation of the ICALM (Interpersonal Counselling for Adolescent Low Mood) feasibility randomised controlled trial
Source: Pilot Feasibility Stud. 2024 Jan 23;10:14. doi: 10.1186/s40814-023-01427-7 (PMC10804551; doi:10.1186/s40814-023-01427-7)
Supplement: Supplementary file 2 — Additional file 2. Characteristics of participating early help and targeted services. [file 40814_2023_1427_MOESM2_ESM.docx]

**Additional file 2: Characteristics of participating early help and targeted services**

|  | **Suffolk** | | | | | | **Norfolk** | | | |
| --- | --- | --- | --- | --- | --- | --- | --- | --- | --- | --- |
|  | **Site-01** | | **Site-02** | **Site-03** | **Site-04** | **Site-07** | **Site-06** | **Site-05** | **Site-08** | **Site-09** |
| **Type of agency** | **Early Help Team** | | **Early Help Team** | **Early Help Team** | **Wellbeing Service** | **Charity** | **Charity** | **Charity** | **Community NHS Trust** | **Family Support Team** |
| **Service provided** | Early help only - non-crisis preventative service | | Early help only - non-crisis preventative service | Early help only - non-crisis preventative services | Targeted mental health service | Targeted mental health service, (independent but also under contract with 04) | Targeted mental health service operating under the Point 1 provision umbrella. Works closely with 05 | Targeted mental health service operating under the Point 1 provision umbrella | Early help only - Emotional Health Team providing support at a Universal Services Level | Family Support team |
| **Number** (or proportion) of YP and/or families with mental health problems in the previous year** | 160-170 YP; close/open about 5-10 referrals per week so potentially 390 families/yr | | 80-85% of referrals have some element of mental health difficulties | Approx. 75% of referrals are around CYP with mental health difficulties | Approx. 1250-1500. 80% of the referrals are for anxiety and depression | Most common presentation is anxiety | Approx. 100. 40-50% of the cases are low mood owing to friendship fallouts, COVID, school avoidance | Approx. 1500 | 90% of referrals associated with anxiety likely impacted by COVID i.e., school avoidance | Approx. 500 open at any one time, a proportion of these children will have mental health needs |
|  | | **Types of mental health difficulties seen (not necessarily treated) by the services** | | | | | | | | |
| **Low mood/** | **Y** | | **Y** | **Y** | **Y** | **Y** | **Y** | **Y** | **Y** | **Y** |
| **Anxiety/stress** | **Y** | | **Y** | **Y** | **Y** | **Y** | **Y** | **Y** | **y** | **Y** |
| **Trauma** |  | |  | **Y** | **Y** |  | **Y** | **Y** |  | **Y** |
| **Abuse** |  | |  |  |  |  |  | **Y** |  |  |
| **Panic attacks** |  | |  | **Y** |  |  |  |  |  |  |
| **Phobias** | **Y** | |  | **Y** | **Y** |  |  |  |  |  |
| **Low confidence** | **Y** | |  |  |  |  |  |  |  |  |
| **Suicidal ideation** |  | |  | **Y** |  |  |  |  |  | **Y** |
| **OCD** |  | |  |  | **y** |  |  |  |  |  |
| **Emotional dysregulation** |  | |  |  |  |  | **Y** |  | **Y** |  |
| **EUPD** | **Y** | |  | **Y** |  |  |  |  |  | **Y** |
|  | | **Types of mental health-related support offered by the services (apart from IPC-A)** | | | | | | | | |
|  | Promotion of positive mental health and wellbeing | | Promotion of positive mental health and wellbeing | Promotion of positive mental health and wellbeing | Webinars on managing anxiety and low mood; Coming out of lockdown. | Low mood – Counselling to enable the development of coping strategies | Low mood and anxiety - CBT informed guided self-help | Low mood – counselling to identify causes/develop coping strategies | Mental health promotion for new emerging mental health difficulties including low mood | Anxiety/ low mood e.g., talking emotions and feelings and developing coping strategies |
|  | Self-identity/esteem | | Trauma Informed Practice |  | CBT informed guided self-help for low mood or anxiety | Emotional wellbeing and resilience building | Processing difficult experiences (mild trauma) |  | Emotional health promotion | Emotional resilience and confidence building |
|  | Confidence & bullying | |  |  | Short term counselling | Youth work with a therapeutic foundation e.g., walk & talk group | Psychoanalytic, trauma informed practice |  |  | In-call service to offer support including mental health support |
|  | Gender & sexuality | |  |  | Behavioural activation |  | Psychosocial intervention |  |  | Wellbeing support |
|  |  | |  |  | Psycho-education |  | Play/talking therapy |  |  |  |
|  | | **Staff characteristics** | | | | | | | | |
| **Staff FTE** | 14.5 | | 20 | 16 | 18.2 | 32 | 12 | 51 | 15 | Approx. 30 |
| **Types of Staff** | - FSP (9.7)  - SFSP (1)  - YPW (1.8)  - PM (1)  - PL (1) | | - FSP (12)  - SFSP (1)  - YPW (3)  - PM (1)  - PL (2) | - FSP (10)  - SFSP (1)  - YPW (3)  - PM (1  - PL (1) | - CWP (5.6)  - PMHW (7)  - CBT (3.8)  - CTM (1.8) | - Counsellors (23)  - Student placements and trainees | - CWP (n/a)  - Counsellors | - Counsellors (n/a)  - PMHW (n/a)) | - Psychologists (2)  - Health visitors (2)  - School nurse (1)  - REHP - (9)  - CWP (1) | FSP  PM |
|  | **FSP** - Family Support Practitioners; **SFSP** - Senior Family Support Practitioner; **YPW** - Young person’s workers; **CWP** - Children’s Wellbeing Practitioner; **CBT** - Cognitive Behavioural Therapist; **PMHW** - Primary Mental Health Workers; **REHP** - Resilience & emotional health practitioners; **PM** - Practice Manager; **PL** - Practice lead; **CTM** - Clinical Team Manager | | | | | | | | | |
| ** Numbers provided by sites are a rough estimate as systems don’t capture historical data so some - figures based on information about live cases and estimated assessments. | | | | | | | | | | |
