# Supplementary material for: Doing research in non-specialist mental health services for children and young people: lessons learnt from a process evaluation of the ICALM (Interpersonal Counselling for Adolescent Low Mood) feasibility randomised controlled trial
Source: Pilot Feasibility Stud. 2024 Jan 23;10:14. doi: 10.1186/s40814-023-01427-7 (PMC10804551; doi:10.1186/s40814-023-01427-7)
Supplement: Supplementary file 3 — Additional file 3. The impact of the COVID-19 pandemic on the services and the ICALM trial. [file 40814_2023_1427_MOESM3_ESM.docx]

**Additional file 3a: The impact of the COVID-19** **pandemic on the services and the ICALM trial**

| **Contextual feature** | **Illustrative quotes** |
| --- | --- |
| **Low mood no longer the**  **most frequent presentation** | “As we’re coming out of COVID, things might change but at the moment we’re seeing lots of anxiety but going forward that might change to low mood” **(SPQ-02, Site-02_Early Help)**  “Anxiety is the major presentation…it’s a problematic thing isn’t it because it is really low mood that is being targeted through IPC. It’s quite unusual to find that presentation of low mood that fits with that right now. Increase in young people who are anxious and not wanting to go out. School refusal is a steadily increasing issue” **(Stakeholder-09, Site-05_Charity)** |
| **Increased (and worsening of) mental health difficulties amongst CYP** | “We became involved with ICALM initially because of the cases we were seeing where wellbeing was one of the main factors, or the major factor. However, since COVID, the need has risen and often the mental health difficulties present in our cases is over and above what the FSP’s can deal with and exceeds the threshold for IPC-A. This was unforeseen really, since COVID specialist MH services have had to become involved with a lot of our cases, whereas, perhaps before coronavirus, they probably would have been OK for IPC” **(SPQ-02, Site-02_Early Help)**  “Our service is meant to be for mild to moderate help requiring ‘short-term, light touch’ interventions however we have seen a huge increase in complexity and risk…we’re seeing more moderate to severe presentations, rather than mild to moderate – young people are presenting with increased suicidal ideation and greater chronicity. We noticed these changes starting in Feb/March 2020 and there was already a capacity/demand issue, but it’s just increased during COVID. We used to be working with green cases, now we rarely see green cases, mainly Amber ones.” **(SPQ-04, Site-04_Wellbeing Service)** |
| **Changes in mode of intervention delivery resulting to variations in CYP engagement** | “No, we hardly did anything online pre-pandemic but now we are finding some young people are preferring this way, not all of them, but some. I think we’ll keep delivering some stuff online, if young people want it and respond positively to this mode of communication and engagement” (**SPQ-03, Site-03_Early Help)**  “The impact of COVID has meant some people could not access treatment as engaging virtually was too difficult for them. Some people who had started treatment chose to pause treatment whilst it was offered virtually and will (or have already) resume once we are undertaking face to face more readily” (**SPQ-04, Site-04_Wellbeing Service)** |
| **Reduced capacity and a higher demand** | “The other major difficulty is that there’s just been such…very high levels of sickness…And obviously… people having to home school and being therefore unable to work because they had children of an age that they couldn’t – they had to look after them basically. So, there were those impacts” **(Stakeholder-09, Site-05_Charity)**  “So, if we’ve got like – OK, we’ve got all these families coming in and then it’s like OK, but we’ve actually got this additional thing that we offer it can feel that sometimes the IPC is an additional thing” **(Stakeholder-10, Site-03_Early Help)** |

**Additional file 3b: – Structural challenges affecting central referral points and frontline services**

| **Macro contextual feature** | **Illustrative quotes** |
| --- | --- |
| **Knowledge of (and support for) frontline** | “In mental health there is such a lack of knowledge and resources at the very, very front line that they just tick a box, they literally have just got to put low mood or ADHD, and that’s it, ping, not my problem. You would not be able to do that in any other health field whatsoever. But yet it’s acceptable in mental health…” **(Stakeholder-07, Site-04_Wellbeing Service)**  “…I’m not sure how often it happens, but the GP’s sometimes ticking the psychosis box for really young children. And I mean the evidence base around young children and psychosis is negligible, isn't it? It’s nothing really. It’s a certain age where presentation’s more common, isn't it, above 14 I think or something” **(Stakeholder-02, Site-04_Wellbeing Service)** |
| **Incomplete referral forms** | “They’ve got hardly any information, things aren’t ticked properly, risk isn’t explained, so they don’t give us an idea of if they’ve given a young person a safety plan, or even spoken to them about any advice and guidance. So, if that’s not included in the referral and we don’t have adequate information, we will then have to call them as well to gain that information... Sometimes self-referrals can be quite tricky, they don’t really know what we want and what information we sort of need providing” **(Stakeholder-11, Site-06)**  “Yes, it’s really easy for the GP to tick a box to say somebody might have ADHD, but the result of that is the hub practitioner has got to do so much digging and unpicking and investigating into that, just from the fact that the GP’s ticked a box [based] on something that someone’s said to them” **(Stakeholder-02, Site-04_Wellbeing Service)** |
| **Improper use of mental health language** | “Sometimes people will say, “I’ve got depression” as opposed to, “I’ve got low mood.” …it’s the language people use, and we try to unpick that…like when people are saying about this person’s got depression. Well, is this like clinical depression? Is this a diagnosis you’ve got? You know, when people have got generalised anxiety, you’re not going to get a seven-year-old that’s diagnosed with that. There might be some anxious traits that you’re seeing…there might be some low mood… and it’s really important trying to get that common language used across the board in all services.” **(Stakeholder-07, Site-04_Wellbeing Service)**  “But what we’ve also found is they’re asking for an ADHD assessment, and it says, ‘ADHD diagnosed’ and has a date. So, we’re like, well why are they diagnosed? No, what it’ll be is a SENCo (Special Education Needs Coordinator) has said to a parent, ‘I think your kid’s got ADHD’ They go and tell the GP. The GP thinks it’s a diagnosis and records it on their record. That cannot be removed, and they may not even have it” **(Stakeholder-01, Central referral point)** |
| **Mental health education and understanding the role of society in mental health** | “I think there’s lots of social media, lots of stuff on TV … you look at Panorama when they do my kid has got ADHD. Good watching, I guess for parents of children with ADHD but not overly helpful to us actually. And when I say us I mean health, everybody, because I think a balanced approach would be to say what are you doing as parents to manage this…What are you doing to get the support and guidance you need rather than pushing on to somebody else to get?” **(Stakeholder-06, Site-09_Family Support Team)**  “In the last two years…there is an ability for some parents to blame… I think we have an increased amount of that. ADHD is a really simple one. What does the label of ADHD get you? For many parents in our service, it gives them that vindication that it’s not their fault. And I don’t think it’s about fault because when you ask health, parents in courses…about parenting kids with ADHD, tell me what we do different, nothing at all. We approach parents exactly the same. So, I think there’s a misconception that if your child gets labelled you get more” **(Stakeholder-06, Site-09_Family Support Team)** |

**Additional file 3c: Mental health policy influencing the ICALM trial at a macro-contextual level**

| **Type of policy, discourse relevant to implementation** | **Description** |
| --- | --- |
| [No Health without Mental Health](https://www.gov.uk/government/publications/the-mental-health-strategy-for-england)^45^ (2011) | Underpinned by a philosophy that “Mental health is everyone’s business – individuals, families, employers, educators and communities all need to play their part.” Strategy focused on early intervention, cross-sectoral collaboration and measurement of outcomes using the NHS Outcomes Framework domains. |
| [Future in Mind](https://assets.publishing.service.gov.uk/government/uploads/system/uploads/attachment_data/file/414024/Childrens_Mental_Health.pdf)^48^ (2015) | The Children and Young People’s Mental Health and Wellbeing Taskforce identified how to improve children  and young people’s mental health services across 5 themes: **1)** Promoting resilience, prevention and early intervention **2)** Improving access to effective support – a system without tiers **3)** Care for the most vulnerable **4)** Accountability and transparency **5)** Developing the workforce |
| [The Five Year Forward View for Mental Health](https://www.england.nhs.uk/wp-content/uploads/2016/02/Mental-Health-Taskforce-FYFV-final.pdf)^49^ (2016) | Taskforce called for recommendations from Future in Mind to be implemented in full. Objective to provide mental health care to 70,000 more children and young people by 2020/21. Waiting times should be substantially reduced, significant inequalities in access should be addressed and support should be offered while people are waiting for care. |
| [Green Paper on children and young people’s mental health](https://www.gov.uk/government/consultations/transforming-children-and-young-peoples-mental-health-provision-a-green-paper)^50^ (2017) | Green Paper proposed: **1)** to create a new mental health workforce of community-based mental health support teams; **2)** to create mental health support teams in schools and colleges. For every school and college encouraged to appoint a designated lead for mental health and every secondary school to be offered mental health first aid training; **3)** 4-week waiting time for NHS children and young people’s mental health services to be piloted in some areas. New trials to look at how to strengthen the links between schools and local NHS mental health staff.  Major thematic review of children and adolescent mental health services across the country, led by the Care Quality Commission, to identify what is working and what is not. By 2020/21, at least 1,700 more therapists and supervisors will need to be trained and employed to meet additional demand, and the strategy will also outline actions needed to improve retention of existing staff. All localities should work with the existing Children and Young People’s Improving Access to Psychological Therapies (CYP IAPT) programme to deliver post-graduate training in specific therapies, leading to at least 3,400 existing children and young people’s mental health service staff being trained by 2020/21. |
| [NHS Long Term Plan](https://www.longtermplan.nhs.uk/)^51^ | Restated the commitment in The Five Year Forward View for Mental Health to improve access to mental health treatment for 70,000 more children and young people and continued expansion of CYP mental crisis services. |
| Office of Health Improvement (formerly PHE)^52^ | Focused on specialist treatment and importance of adding capacity to specialist treatment teams and reducing waiting lists across specialist services. |
| Status of mental health of children and young people in July 2020, during the Coronavirus (COVID-19) pandemic and changes since 2017^53^. | Rates of probable mental disorders increased from one in nine (10.8%) children aged 5 to 16 years in 2017 to one in six (16.0%) in 2020.  The likelihood of a probable mental disorder increased with age with a noticeable difference in gender for the older age group (17 to 22 years); 27.2% of young women and 13.3% of young men were identified as having a probable mental disorder. |

**Additional file 3d: Meso-contextual features of participating services and their impact on the ICALM trial**

| **Meso-contextual features** | **Description** | **Illustrative quotes** |
| --- | --- | --- |
| **Increased complexity of cases** | | |
| **Cases no longer meeting service early help thresholds** | Long waiting times for green cases led to increased complexity of cases and CYP no longer meeting services thresholds. This was a key barrier to identifying appropriate cases for the ICALM study. | “There’s some fairly huge waiting lists for young people that are those types of referrals that you’re talking about…the kind of cases that would just sit on this waiting list… waiting for some sort of mental health service to come about. And it never comes. They never reach the threshold for any sort of therapeutic intervention unless it’s much, much more serious.” **(Stakeholder-04, Site-01_Early Help)**  “There are lots of difficulties at the moment with the Central referral point. They have a waiting list of 2000 young people who have been RAG Rated as Green and the minimum wait for Green cases is 100+ days for any kind of service. They’re looking at those young people that have gone over 100 days and whether they can come to Early Help. But their green is not necessarily going to be my green. At the moment, I’m not taking any, quite rightly, as they’re not appropriate” **(SPQ-02, Site-02_Early Help)** |
| **Increased complexity of cases referred to early help creating a huge demand for targeted services** | Despite acknowledging mental health as the biggest underlying need for supporting CYP, the current inability for early help to take the lead in providing mental health support, has created a huge demand for targeted services which are already under pressure. | “Mental health is one of our biggest, if not the biggest underlying need for supporting children and young people. And the gaps are that we can’t always access the relevant services as quickly as we’d like to. So, for example, we might do a referral to the Suffolk Wellbeing Hub, but the turnaround might be 12 weeks and then often what happens is people then come back to our service, but we were the original referrers, and not enough support from mental health or special education needs services ” **(Stakeholder-10, Site-03_Early Help)**  “And I guess sometimes historically there’s been quite a wait for [a service] so when we feel we need to escalate a child or young person sometimes they get told there’s quite a wait. And there’s always that debate around whether or not it would be appropriate for us to offer some low-level psycho education type strategies whilst there’s a wait and sometimes that is appropriate, and sometimes it’s not because of the level of risk or the level – particularly low mood, potentially if the person’s motivations change its maybe impacted then actually it needs a higher tier than us” **(Stakeholder-03, Site-08_Community NHS Trust)** |
| **Increased complexity of cases referred to targeted services creating a huge demand for Tier 3 services** | Cases referred to targeted services are also getting more complex, with most requiring escalation to tier 3 services. Apart from the huge demand for tier 3 services, targeted services also have challenges in setting up interface meetings with other services to discuss the cases. | “Because there are long waits. So, for example, if we wish to at the moment refer someone to the [Tier 3] there is approximately a year’s wait for a routine assessment. So, this is the main challenge… I’ve referred a young person in October for a routine assessment and I was told that they might get seen in June if I was fortunate, but it might be this October. And of course, having a routine assessment doesn’t mean to say you’ll be accepted for an intervention, and if you are accepted for an intervention, you are then going to have to go on an intervention wait list” **(Stakeholder-09, Site-05_Charity)**  “So, we have daily meetings, or daily interface meetings, with tier 3 services. So, let’s say there was a quite complex case with a lot of trauma, that same day or maybe the next day, I could have a conversation with the service in their area and we would discuss the case and see if they would be able to provide support as well. So, the communication is improving between services, which is good” **(Stakeholder-11, Site-06_Charity)** |
| **Service specifications and referral pathways** | | |
| **** Mental health support not the central focus for early help interventions** | Despite a large proportion of referrals consisting of mental health difficulties, the early help offer is not mental health focussed. This means that early help teams do not take a lead on mental health issues but work alongside or refer cases to targeted services who take the lead where there is a mental health concern. | “I don’t even think I could give you a ballpark figure. But recent stats have shown that approximately three quarters of our referrals are around young people’s mental health.” **(SPQ-03, Site-03_Early Help)**  “We would work alongside any families experiencing any mental health problem, we wouldn’t necessarily take the lead on the mental health part...It is common for us to have Family Plans that include an element of mental health concern, but in the cases where this is more complex, we would be working alongside more specialist mental health services in the spirit of multi-agency working, and hope that the person is accessing therapy or treatment. The thing we try and avoid is other services stepping away because Early Help are involved” **(SPQ-01, Site-01_Early Help)** |
| **Gaps in interventions provided by tier 2 and 3 services** | There are treatment gaps between targeted services and tier 3 services whereby some young people require longer term interventions (targeted services deliver short term interventions) but don’t reach the criteria for tier 3 services. There are also come gaps whereby for some conditions there is no available support either in primary or secondary care. | “So that’s a real, that’s a challenge for us, because it’s not quite reaching the criteria of secondary, because there isn't the risk. Sometimes there is the risk and we can step up straightaway, but often there isn't the risk but there is definitely the complexity. Secondary are also overrun and struggling and they’re almost crisis managing. So their criteria have increased. So the bottom of their criteria, which would not meet our criteria as such, we’re almost rising up to meet that because there’s nowhere else for these people to go, but it’s not short-term primary intervention. That’s just not what it is.” **(Stakeholder-07, Site-04_Wellbeing Service)**  “What we tend to do is if we’ve got cases and we’ve tried to step them up and secondary have said no, we’ve got a meeting that we have called patient flow, and it was set up for those cases that get stuck in between teams. We’re saying it’s too complex for us and secondary are saying it’s not complex enough for them or it’s not risky enough, then we take, we present those cases. Then somebody, usually one of the senior managers or somebody above us will say, actually it needs to sit with you, it needs to stay with your team, or it needs to go to this team. They make that decision” **(Stakeholder-07, Site-04_Wellbeing Service)** |
| **Out-dated or unclear service specifications** | As the demands for services rise and the mental health system experiences pressures at every level, service specifications are appearing outdated or unclear  This knock-on effect has put a lot of pressure on early help and targeted service providers, with some reporting having to deliver ‘holding interventions’ or update their service specification in order to provide support to CYP whilst they wait for the right services. | “So most teams have got like a service spec, but our service spec is really outdated…So we’ve just ended up holding a lot of these cases, as I’m sure secondary have held cases that probably their team wouldn't normally take. It would go to a crisis team” **(Stakeholder-07, Site-04_Wellbeing Service)**.  “I think our service spec is only considered out of date because of the pressures on the system. I feel like it’s structured as an early intervention service spec…but we’ve just morphed into a service that adapts and accepts that we need to extend our boundaries a little bit if you know what I mean. It is an early intervention service spec but there’s nowhere else for these patients really to go into the system at the moment” **(Stakeholder-02, Site-04_Wellbeing Service)**  “We’ve had so many senior managers try and define what early help actually offer – but it’s such a broad service, it’s really difficult to do. There are 13 locality teams – all do things slightly differently; they have different local partnerships and services and may support families in a variety of different ways. We address lots of the systemic difficulties, all of which will have a knock-on impact on the mental health of young people. It’s easier to list what we don’t do, rather than what we do, do. We’ve become a jack of all trades due to all the difficulties families can present with” **(SPQ-01, Site-01_Early Help)**  “Sometimes we're not the right offer for young people and we wish to refer them to see a psychiatrist or psychologist but know they're going to wait a year for an assessment, that's a massive challenge. What do we do with that young people when we already know there's a need? Come to an end of an interventions with young people and want to refer on - routine assessment may take a year and does not mean will be accepted for an intervention, and if are accepted, then have to go on intervention wait list” **(Stakeholder-09, Site-05_Charity)** |
| **A system or management level dysfunction** | A system level dysfunction driving the lack of clarity of treatment pathways between services and causing delays in treatment | “Well, I mean some of it is a demand but as I say some of it is just disorganisation…Ten years ago, I asked what the pathways were within (the mental health Trust) that we could refer to. To this day nobody has written down what the pathways are. So, when I’m working with a young person it’s really difficult for me to know what other services there are for them to be referred to. I know there’s a range of different professionals within (the mental health Trust), but I don’t know, for example, if there’s a pathway that’s for acceptance commitment therapy. I don’t know whether there’s one person delivering that therefore it’s unreasonable to think that anyone is going to get that, or whether there’s a team of ten” **(Stakeholder-09, Site-05_Charity)**  “It’s impossible to know the realistic options, yes, because…for some reason…there’s been block contracts in which there’s not been a clarity about the resourcing of the trust I think it’s fair to say, and what exactly they’re doing with the money that they’ve got” **(Stakeholder-09, Site-05_Charity)**  “A lot of the time it’s driven by process and system issues, and the fact that services are overstretched and they’re trying to move people on a lot of the time, and not rightly or wrongly, but I would say that that’s a huge part of it as well” **(Stakeholder-01, Central referral point)** |
| **Capacity and funding** | | |
|  | Both early help and targeted services are facing capacity challenges, which have been exacerbated by the COVID-19 pandemic.  Capacity challenges, including staff turnover, have had a significant impact on recruiting into ICALM as most services were left with a limited number of staff who were able to deliver IPC-A | “But for me it’s about resourcing and numbers, you know, and I have a team of 12 people which isn’t huge for the whole of Bury St Edmunds, and like I said earlier 186 something cases" **(Stakeholder-04, Site-01_Early Help)**  Cases were identified through the allocated workers and the managers allocating the cases. So, there was a huge burden of responsibility on the poor managers. One of those managers moved on. So, that was down to three. One of those managers took an acting job so that was down to two managers. That was about replacing those managers, but I guess we were so far down the line that never happened. **(Stakeholder-06, Site-09_Family Support Team)** |
